# Exploiting volume electron microscopy to investigate structural plasticity and stability of the postsynaptic compartment of central synapses

**DOI:** 10.3389/fncel.2023.1153593

**Published:** 2023-03-24

**Authors:** Greta Maiellano, Lucrezia Scandella, Maura Francolini

**Affiliations:** ^1^Department of Medical Biotechnology and Translational Medicine, Università degli Studi di Milano, Milan, Italy; ^2^MeLis, CNRS UMR 5284, INSERMU1314, Institut NeuroMyoGène, Université de Lyon, Université Claude Bernard Lyon 1, Lyon, France

**Keywords:** volume electron microscopy (vEM), postsynaptic density (PSD), structural plasticity, dendritic spine enlargement, axon-spine interface, synaptic apposition surface, synapse, synaptic stability

## Abstract

Volume reconstruction from electron microscopy datasets is a tool increasingly used to study the ultrastructure of the synapse in the broader context of neuronal network and brain organization. Fine modifications of synapse structure, such as activity-dependent dendritic spine enlargement and changes in the size and shape of the postsynaptic density, occur upon maturation and plasticity. The lack of structural plasticity or the inability to stabilize potentiated synapses are associated with synaptic and neuronal functional impairment. Mapping these rearrangements with the high resolution of electron microscopy proved to be essential in order to establish precise correlations between the geometry of synapses and their functional states. In this review we discuss recent discoveries on the substructure of the postsynaptic compartment of central excitatory synapses and how those are correlated with functional states of the neuronal network. The added value of volume electron microscopy analyses with respect to conventional transmission electron microscopy studies is highlighted considering that some limitations of volume-based methods imposed several adjustments to describe the geometry of this synaptic compartment and new parameters–that are good indicators of synapses strength and activity–have been introduced.

## Introduction

Our brain discards old and useless information in favor of new and more important ones thanks to one of its amazing properties: synaptic plasticity. Synaptic plasticity underlies the ability to respond to activity-dependent stimuli (Citri and Malenka, 2008; Goto, 2022). The neuronal activity generated by any experience directly modifies the neural circuit by remodeling synaptic transmission itself, which is made possible by the release of neurotransmitter from the pre- to the postsynaptic terminal. Activity-dependent changes occur structurally at the level of the synapse, either by formation of new synapses or by modification of pre-existing ones, and, therefore influence how we think, feel and behave. Those structural changes affect subsynaptic structures such as the active zone (AZ) in the presynaptic compartment as well as the postsynaptic density (PSD) and dendritic spines in the postsynaptic neuron. These different structures are highly correlated in their size, so their dimensions are often used as a measure of the activity and strength of the synapse.

The notion that alterations in the structure, size and strength of the synapse might be at the basis of important processes like neural development, learning and memory is quite straightforward, as is the idea that an aberrant synaptic plasticity might be connected to pathological conditions (Borczyk et al., 2021). Indeed, alteration and/or disruption of neural circuits can have huge consequences on synapse structure and dynamics as demonstrated by their involvement in diverse pathological conditions such as motor and learning disabilities, altered mental status, cognitive impairment, neurodegenerative diseases (Chidambaram et al., 2019; Borczyk et al., 2021).

These considerations have motivated studies to elucidate how and to what extent synaptic structures change during synaptic plasticity. The use of confocal and two-photon (2P) excitation microscopes, together with the use of fluorescent probes and/or the expression of an ever-growing number of fluorescent proteins and biosensors in living neurons, is an important tool to study synapse structure and dynamics of both *in vitro* and *in vivo* systems, but, due to the limited spatial resolution of optical microscopes, they might fail to detect many of the subtle changes that characterize synaptic structure and plasticity.

## Volume electron microscopy techniques in the study of synapses

The ability of the Electron Microscope (EM) to achieve nanometric resolution makes it the best instrument to visualize synapse ultrastructure. However, when visualizing individual Transmission Electron Microscope (TEM) bidimensional projections, the variability related to the plane of the sections and the lack of information on the *z*-axis strongly reduce the amount of data that can be collected from each image (Colombo et al., 2021). These limitations can be overcome to some extent by volume Electron Microscopy (vEM). Volume EM includes all EM methods able to generate a continuous series of bidimensional projections from resin-embedded samples with a depth greater than 1 μm (Peddie et al., 2022). Among vEM techniques TEM based approaches are included, such as serial section TEM (ssTEM) and serial section Electron Tomography (ssET), as well as techniques based on the use of the Scanning Electron Microscope (SEM) to visualize the face of the embedded tissue block by means of the backscattered electrons. The use of the SEM is coupled to the possibility to iteratively section the block as in the serial block face-SEM (SBF-SEM), or to mill out small portions of its surface (focused ion beam-SEM or FIB-SEM) in the SEM chamber. An alternative approach still taking advantage of the SEM backscattered electrons is represented by the array tomography in which long series of ultrathin sections collected on different substrates (coated glass slides, silicon wafers or tapes) are visualized under the SEM electron beam. These methods allow the experimenters, by analyzing three-dimensional vEM datasets, to obtain information on tissues and cells ultrastructure on the *x*, *y*, and *z* axes. These different techniques, although having different spatial resolution (indeed they generate near TEM-quality ultrastructural data but still they do not reach TEM spatial resolution in the *x*, *y* axes) produce datasets from large volumes of tissue allowing for the segmentation and reconstruction of the structure under investigation (Titze and Genoud, 2016; Kubota et al., 2018; Peddie et al., 2022). Even if we recently reported that some relevant aspects of the postsynapse architecture can be faithfully evaluated from TEM bidimensional projections (Colombo et al., 2021), measures from reconstructed SEM-based vEM stacks proved to be the favorite choice when dealing with the analysis of postsynaptic organization and its structural plasticity. The major limitations of these vEM approaches are represented by the large amount of time and computational work needed to analyze volumetric dataset and the paucity of tools able to recognize and segment the object of interest in a fully automated manner.

## The ultrastructure of a chemical synapse

Since different physiological and pathological conditions are associated with modifications of synaptic components (Chidambaram et al., 2019), many studies in recent years aimed to define precise parameters to describe its ultrastructure, allowing for detailed and quantitative comparisons. The chemical synapse is a specialized intercellular junction that permits communication through neurotransmitter release between a pre- and a postsynaptic neuron, divided by a thin intercellular space, the synaptic cleft. The efficacy of a presynaptic terminal can be ultrastructurally defined by the size of the presynaptic bouton and by the amount, density and distribution of the synaptic vesicles in the bouton (Cheetham et al., 2007). Vesicles are docked and released at the presynaptic active zone (AZ), a dense meshwork of proteins involved in vesicle docking, priming and fusion (Südhof, 2012). The AZ, in mammalian central synapses, is occasionally visible with TEM as thin electron dense protrusions beneath the plasma membrane (Ackermann et al., 2015). Conversely, the postsynaptic component of the synapse is characterized by the presence of numerous proteins embedded in its membrane and just beneath it, involved in neurotransmitter binding (membrane receptors) and in the intracellular signaling pathway triggered in response to receptor activation. In EM micrographs, these clusters of proteins are detectable as an electron-dense flattened structure, the postsynaptic density (PSD), which can be described in terms of length, thickness and volume (Murru et al., 2017; Longaretti et al., 2020; Vezzoli et al., 2020). The PSD is thicker in excitatory synapses than in inhibitory ones (Tao et al., 2018), and in the case of excitatory synapses the PSD is most often localized on a tiny protrusion of the dendrite, the spine.

## Postsynaptic density, synaptic apposition surface (SAS) and axon spine interface (ASI)

In recent years, vEM confirmed an observation about the geometry of the PSD that was already reported from TEM analyses of central synapses (Toni et al., 2001; Morales et al., 2011 and references therein); the PSD is not always a simple discoidal structure whose shape can be simply described with length, thickness and volume, but instead it can undergo morphological changes, which reflect different functional states of the synapse (Hering and Sheng, 2001). To use the geometry of this important component of the postsynapse to quantitatively characterize neuronal connectivity across large portions of the neuropil, huge vEM dataset were generated, and the need to analyze this large amount of data led to the development of automatic software for the segmentation and reconstruction (Morales et al., 2011; Cardona et al., 2012; Berg et al., 2019; Zumbado-Corrales and Esquivel-Rodríguez, 2021), which, in some cases reduced the spatial accuracy of the analysis.

Through FIB-SEM studies several aspects of synapse architecture were defined on reconstructed volumetric datasets. Among those parameters, the synaptic apposition surface (SAS) was defined as the area of close apposition between the membrane of the presynaptic AZ and the membrane of the postsynaptic neuron covering the PSD (Morales et al., 2013). The SAS was introduced as a method to overcome the intrinsic difficulties in identifying, in an automated manner, individual AZs and PSDs that in EM bi-dimensional projections can be sectioned in unfavorable planes (i.e., not perpendicular to the synapse major axis). Since then, the size of the SAS, obtained from vEM datasets, has been considered as a direct indicator of the function and the strength of a synapse since it takes into account features of the pre- and the postsynaptic compartments (i.e., the surface area of the AZ is related to the number of docked vesicles and probability of release, while the size of the PSD as indication of the amount of clustered neurotransmitter receptors). Considering the size, complexity and curvature of the SAS and their relation with the function of the synapse and of the neural network (Santuy et al., 2018), recent studies investigated the alterations in central synapses in Alzheimer’s Disease (AD) patients and rodent models: SAS was reduced in human CA1 (Montero-Crespo et al., 2020, 2021) and rat pre-frontal cortex where the PSD volume was equally modified (Jiang et al., 2022). Other reports highlighted an overall loss of excitatory synapses in the hippocampus of patients and mouse model of AD (Neuman et al., 2015) or their mislocalization on dendritic shafts in the transentorhinal cortex of AD patients (Domínguez-Álvaro et al., 2019), in both cases, however, a marked increase in the percentage of perforated or fragmented excitatory synapses was noted.

Through SBF-SEM another parameter was established, the axon spine interface (ASI), defined as the interfacing surface between the presynaptic bouton and the spine head (Bellesi et al., 2015) and it represents the 3D equivalent of the length of the synaptic cleft which can be observed and accurately measured in TEM images (Colombo et al., 2021). The ASI and the PSD size were shown to be strongly correlated as they both become larger upon synaptic potentiation (Cheetham et al., 2014; De Vivo et al., 2017) and both correlated with the amplitude of excitatory postsynaptic currents (Murru et al., 2017; Nagai et al., 2021). Indeed, the tight relationship between the surface area of the ASI and the functional state of the synapse was demonstrated in a number of studies exploring the effects of sleep and sleep deprivation in homeostatic scaling of neural circuits. These studies showed that synaptic potentiation was associated with enlargement of the ASI in a subset of synapses when the animals were exposed to learning tasks during the wake period, while it underwent renormalization during sleep, possibly to consolidate and integrate memories and to avoid circuit saturation (Cirelli and Tononi, 2020). In fact, it has been shown that sleep induced reduction of the ASI both in hippocampal CA1 and primary motor cortex neurons (De Vivo et al., 2017); accordingly, chronic sleep deprivation led to ASI expansion in hippocampal CA1 (Spano et al., 2019; Nagai et al., 2021). Together with a reduction of the ASI, AMPA glutamate receptor (AMPAR) expression at the postsynaptic membrane was reduced after sleep (De Vivo et al., 2017; Miyamoto et al., 2021); and, in line with these findings, the levels of AMPAR and its phosphorylation were shown to diminish during sleep through a mechanism dependent on the increased synaptic concentration of Homer1a (Diering et al., 2017).

## Dendritic spines size and shapes

Most excitatory synapses in mammalian neurons are present on dendritic spines, small protrusions which permit the electric and chemical compartmentalization of synaptic input. These dynamic structures vary in shape and size, features which are highly dependent on the maturity and functionality of the synapse itself (Berry and Nedivi, 2017): they originate as dendritic processes, highly dynamic and immature, which rarely form a synapse and, after maturation, they acquire a more complex shape, with a thin neck of various length, and a bulbous head, where the synapse is more frequently established (Yuste and Bonhoeffer, 2004). The morphology and plasticity of dendritic spines has long been studied taking advantage of fluorescence imaging but, given their small size, their shape and volume are optimally studied with vEM (Parajuli et al., 2020; Colombo et al., 2021; Parajuli and Koike, 2021).

## Plasticity and its effects on the architecture of the postsynaptic compartment

Several studies have reported a direct correlation between the amount of AMPAR, PSD area and dendritic spine size and shape (Cheetham et al., 2014; Colombo et al., 2021), but several notable exceptions were recently reported (see below). The ability of subsynaptic structures to undergo changes after synaptic stimulation has been explored taking advantage of several models and techniques. Using 2P glutamate uncaging and time-lapse imaging together with TEM, a rapid (within few minutes) enlargement of dendritic spines was observed in rat and mouse hippocampal slices (Bosch et al., 2014; Meyer et al., 2014); interestingly, this enlargement was coupled to a delayed (1 to 3 h) enlargement of the PSD. This enlargement was either persistent (in stabilized spines) or transient and associated with neither increased levels of PSD-95 nor with increased PSD (Meyer et al., 2014). Upon induction of chemical long term potentiation (LTP) in the same mouse model, the increase in spine and PSD size was tightly correlated in small and large dendritic spines, whereas in medium-sized spines the changes in the two structures were independent from each other (Borczyk et al., 2019).

The ability of a synapse to undergo structural changes after synaptic stimulation was also dependent on the presence of membrane-bound intracellular organelles in the spine (Borczyk et al., 2019). Indeed, the presence of the spine apparatus, a specialized compartment of the smooth endoplasmic reticulum found in a subset of dendritic spines, considerably increased the capacity of the synapse to undergo structural changes in neurons in the mouse hippocampus (Perez-Alvarez et al., 2020) and motor cortex (Hedrick et al., 2022). Even if the precise function of the spine apparatus remains elusive, its involvement in LTP-related processes through modulation of calcium dynamics and transport of locally synthesized proteins has been proposed (Jedlicka et al., 2008 and references therein). However, this relation between the presence of endoplasmic reticulum and the ability of the synapse to undergo plastic changes was not exclusive, as in mice primary motor and somatosensory cortex, the presence of recycling endosomes, involved in receptor trafficking (Cooney et al., 2002), has been shown to positively impact on the ability of a synapse to undergo scaling down during sleep (De Vivo et al., 2017).

**FIGURE 1:**
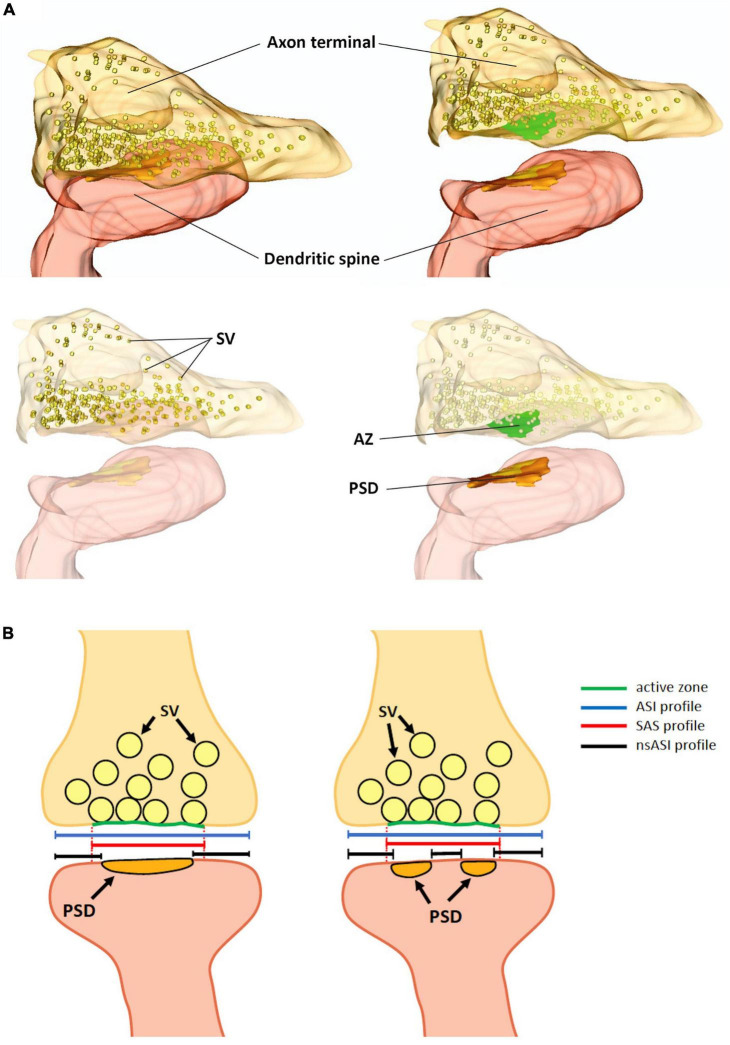
**(A)** Three-dimensional reconstruction of an excitatory synapse, in which the axon terminal, filled with synaptic vesicles (SV) and the active zone (AZ) are visible. In the dendritic spine, the postsynaptic density (PSD) is shown. **(B)** Schematic representation of an excitatory synapse with a macular PSD (left panel) and with a perforated/fragmented PSD (right panel). Dotted red lines are the projection of the active zones on the postsynaptic membrane enclosing the PSD, which define the profile of the synaptic apposition surface (SAS, continuous red line). The axon-spine interface (ASI, blue line), also corresponds to the synaptic cleft length. In the ASI we can distinguish a non-synaptic-ASI (black lines) and a synaptic ASI. The PSD length in mouse hippocampal CA1 pyramidal neurons ranges from 80 to 500 nm; the diameter of the SAS from 80 to 600 nm and the synaptic cleft, (the ASI) could be as long as 700 nm. SV, synaptic vesicles; AZ, active zone; PSD, postsynaptic density.

Finally, by using 2P glutamate uncaging to promote structural LTP of individual spines in mouse hippocampal organotypic slices, it was shown that a fast dendritic spine enlargement (within 2–3 min from stimulation) was paralleled by PSD perforation and segmentation but not with a concomitant increase in the PSD surface area, suggesting that the increase of the ASI was not faithfully explained by PSD enlargement and introducing the concept of the non-synaptic ASI (*nsASI*) whose surface increases faster than the *canonical* ASI (that is equal to *nsASI* + PSD area) (Sun et al., 2021) (Figure 1B).

Thus, while PSD, SAS, and ASI identify different portions of the synapse (Figures 1A, B), with ASI and SAS being broader than the PSD, these parameters are strongly correlated and they all correlate, to a variable extent, with the spine head volume (Colombo et al., 2021).

## Concluding remarks

The study of synapses in two dimensions, as allowed by single section TEM imaging, implies the loss of spatial information that can be retrieved only in three dimensions with vEM (i.e., PSD complexity and dendritic spine shape) (see Colombo et al., 2021). For this reason, SEM-based vEM techniques are now privileged in studies addressing synaptic structural plasticity and stability on relatively large volumes of the neuropil. None of these techniques, however, offers the same spatial resolution on the *x* and *y* axes of TEM, not only due to intrinsic imaging limitations, but also for the necessity to heavily infiltrate samples with metals in order to render them conductive and to achieve adequate backscattered electron contrast. The enhanced contrast is needed to facilitate the automated or semi-automated recognition of the structures of interest. In those datasets it was often challenging to distinguish between the synaptic and non-synaptic portions of the postsynaptic membrane and so it was necessary to introduce new parameters to define synapse geometry. These limitations in lateral resolution can affect to different extent SBF-SEM and FIB-SEM images and indeed it was demonstrated that in FIB-SEM datasets measuring the SAS was easier and more reliable than measuring PSD surfaces (Morales et al., 2013). Importantly, these newly introduced elements describing subsynaptic portions that differ from the PSD itself, were demonstrated to be good predictors of synapse strength and activity (Bellesi et al., 2015; Miyamoto et al., 2021).

## Author contributions

GM, LS, and MF contributed to the design of the review, searched and collected all publications relevant to the topic and wrote the first draft of the manuscript. All authors contributed to the manuscript revision, read, and approved the submitted version.
